# A super-oscillatory step-zoom metalens for visible light

**DOI:** 10.3762/bjnano.13.101

**Published:** 2022-10-28

**Authors:** Yi Zhou, Chao Yan, Peng Tian, Zhu Li, Yu He, Bin Fan, Zhiyong Wang, Yao Deng, Dongliang Tang

**Affiliations:** 1 Key Laboratory of Optoelectronic Technology and Systems (Chongqing University), Ministry of Education, School of Optoelectronic Engineering, Chongqing University, 174 Shazheng Street, Shapingba, Chongqing 400044, China; 2 Sichuan Jiuzhou Electric Group Co., Ltd, Mianyang 621000, China; 3 School of Mechanical Engineering, Sichuan University, Chengdu, Sichuan, 610065, China; 4 State Key Laboratory of Optical Technologies for Micro-fabrication, Institute of Optics and Electronics, Chinese Academy of Sciences, Chengdu, Sichuan 610209, China; 5 The Key Laboratory for Micro/Nano Optoelectronic Devices of Ministry of Education & Hunan Provincial Key Laboratory of Low-Dimensional Structural Physics and Devices, School of Physics and Electronics, Hunan University, Changsha 410082, China

**Keywords:** geometric phase, phase-change material, step-zoom lens, super-oscillatory

## Abstract

In recent years, the super-oscillation method based on the fine interference of optical fields has been successfully applied to sub-diffraction focusing and super-resolution imaging. However, most previously reported works only describe static super-oscillatory lenses. Super-oscillatory lenses using phase-change materials still have issues regarding dynamic tunability and inflexibility. Therefore, it is vital to develop a flexible and tunable modulation approach for super-oscillatory lenses. In this paper, we propose a super-oscillatory step-zoom lens based on the geometric phase principle, which can switch between two focal lengths within a certain field of view. The designed device consists of nanopillars with high efficiency of up to 80%, and the super-resolution focusing with 0.84 times of diffraction limit is verified by the full-wave simulation. The proposed method bears the potential to become a useful tool for label-free super-resolution microscopic imaging and optical precision machining.

## Introduction

Due to the diffraction limit, conventional optical imaging systems are unable to surpass a theoretical resolution of 0.5 λ/NA, where λ is the wavelength and NA is the numerical aperture [1]. Super-resolution optical imaging is of significant scientific and application value, which may lead to a revolution in various fields, such as optical microscopy, optical remote sensing, subwavelength lithography, and ultra-high-density data storage. Thus, overcoming the barrier of diffraction limit and achieve super-resolution optical imaging has become a hot topic in the research field of optics.

In recent years, a variety of super-resolution optical microscopy techniques have been developed. For instance, stimulated emission depletion microscopy (STED) can realize the localization of single fluorescent molecules with 1 nm accuracy [2], albeit with the disadvantages of required fluorescence labeling and slow image reconstruction. Super-resolution microscopy based on structured light illumination (SIM) can realize a spatial resolution of λ/5 [3]. However, it requires additional designed illumination patterns and image reconstruction. Near-field scanning optical microscopy can achieve super-resolution imaging by detecting surface evanescent fields of objects [4]. Near-field focusing lenses [5] based on surface plasmons can reach a spatial resolution of 22 nm, but the imaging range is limited to the sample surface, causing difficulties in biomedical imaging. Although negative refractive superlenses and hyperbolic metamaterials [6–7] have been experimentally verified for super-resolution imaging, they exhibit high optical loss and are not suitable for far-field imaging. As a result, it is still a huge challenge to achieve unlabeled far-field imaging without image post processing.

Optical super-oscillation is a unique optical phenomenon in which an optical signal can oscillate faster locally than its maximum Fourier frequency in an optical field with low spatial frequency [8]. In principle, there is no theoretical limit of resolution for the super-oscillation field, which provides a novel way to overcome the diffraction limit and realize far-field super-resolution focusing and imaging. Lately, super-oscillatory lenses, through fine modulation of the amplitude and phase of the optical field, have been used in super-resolution imaging [9–11], heat-assisted magnetic recording [12], and optical metrology [13]. In order to form the specific super-oscillatory optical field, common super-oscillatory lenses usually employ simple binary amplitude and binary phase modulation [14–15] to modulate the incident optical field, thus realizing a specific coherent superposition of the output optical field. This modulation method is relatively simple and accessible for fabrication; however, it significantly weakens the device’s ability to modulate the optical field effectively, which, in turn, limits the performance of super-oscillatory lenses (e.g., efficiency and large sidelobe). The development of metasurfaces has provided an effective approach to modulating amplitude, phase, and polarization of the optical field at the subwavelength scale [16–22]. Compared with metallic metasurfaces, all-dielectric metasurfaces are characterized by high amplitude transmittance, which is important for super-oscillatory lenses with comparatively low focusing efficiency. Generally, for all-dielectric metasurfaces, the larger the refractive index of the material is, the higher the efficiency will be. Meanwhile, a material with a high refractive index is also beneficial to reduce the aspect ratio of a metasurface device. Therefore, for various optical wavebands, a medium with a high refractive index and low extinction coefficient is usually preferred to be the structural material for super-oscillatory lenses. Nonetheless, most of the previous works only describe invariant super-oscillatory lenses, which only work in a limited number of situations. Researchers have demonstrated achromatic super-resolution focusing at several wavelengths with specifically designed amplitude masks. To achieve a broader waveband, the phase modulation of a super-oscillatory lens is divided into two parts: a super-oscillatory phase with a value of 0 or π from a metasurface and a focusing phase from a commercial achromatic lens [14–15]. However, the focal length of those super-oscillatory lenses is fixed. When super-oscillatory lenses are applied in microscopic imaging, the spatial resolution can be improved a lot, while the field of view decreases proportionally. Under these circumstances, it can be hard to locate the target rapidly within the field of view after switching to the super-oscillatory lens. Although dynamically tunable super-oscillatory lenses could be realized by utilizing phase-change materials [23], the problem of inflexibility still exists.

Here, we propose a super-oscillatory step-zoom lens (SSL) that enables super-resolution focusing with two working modes corresponding to different focal lengths. The designed SSL is composed of a fused silica substrate sandwiched by two metasurfaces based on the geometric phase principle. The optical powers with opposite sign of the front and back metasurfaces can be switched by controlling the polarization state of the incident light. The metasurfaces in our SSL consist of high-aspect-ratio nanopillars with different orientations, which can generate the desired super-oscillation light field with high efficiency. The performance of the proposed SSL is analyzed by electromagnetic simulations to verify the super-resolution focusing capability corresponding to two different focal lengths. Additionally, with our method, the focal plane can be changed by switching the polarization of the incident light instead of moving the lens. We believe this unique property bears a great potential to applied in super-resolution microscopic imaging system.

## Design of the Super-Oscillatory Zoom Lens

Similar to previous works [24–26], our proposed SSL can also be designed in two steps: first, a double-layer step-zoom metalens with diffraction-limited imaging performance is designed; second, a super-oscillatory phase is optimized and then superimposed on the stop pupil plane (i.e., the front surface) of the double-layer step-zoom metalens to realize sub-diffraction step-zoom imaging. For the proof-of-concept example, stop aperture, working wavelength, focal length, and field of view (FOV) of the SSL are 20 μm, 632.8 nm, 20 or 40 μm and 3.2°, respectively.

**Figure 1:**
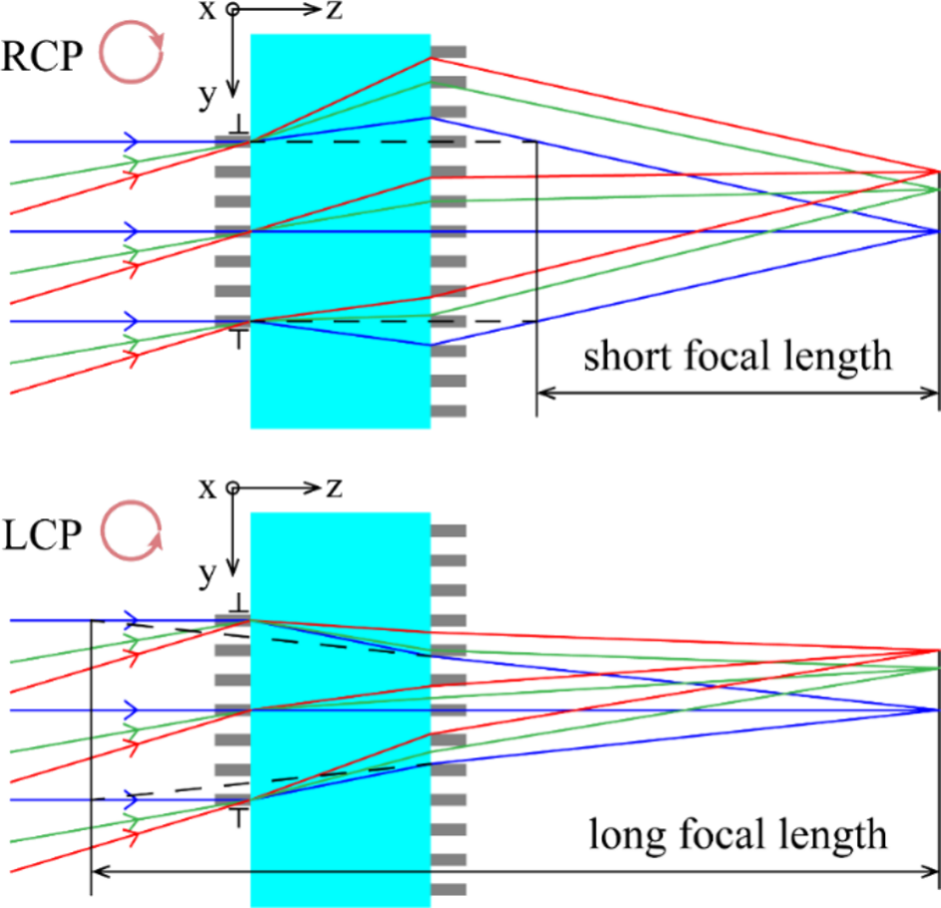
The layout of the double-layer step-zoom metalens.

Essentially, the designed double-layer step-zoom metalens can be regarded as a metasurface doublet cemented by a glass substrate. As illustrated in Figure 1, based on the geometric phase principle, the double-layer step-zoom metalens can be switched between two different focal lengths by controlling the handedness of the incident circularly polarized light. It is superior to the traditional zoom lens since there is no mechanical movement and the image plane of the double-layer step-zoom metalens keeps unchanged. With the geometric optics theory [27], one can get


[1]

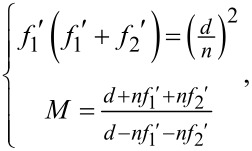



where 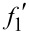 and 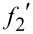 are the focal lengths of front and rear metasurfaces, *d* and *n* are the thickness and refractive index of the glass substrate, and *M* is the zoom ratio. Here, we choose fused silica glass (the refractive index is about 1.457 at a wavelength of 632.8 nm) as the substrate material. *M* and *d* are set to be 2 and 4.86 μm, respectively. The substrate thickness is optimized by ZEMAX for the best optical performance. Certainly, for the practical application, there are more realistic factors that need to be considered. It is worth noting that there is always a tradeoff between performance and practical conditions. 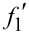 and 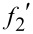 were calculated to be ±10 μm and ±8.9 μm. In this way, a double-layer step-zoom lens has been obtained by combining two metasurfaces with opposite optical powers. In order to correct the optical aberration more effectively, we use the even aspherical phase profile rather than the parabolic phase profile for the phase modulation in these two metasurfaces, which can be denoted as:


[2] $$\varphi \left(r\right)=\sum_{i=1}^{n}a_{i}{\left(\frac{r}{R}\right)}^{2i},$$

where *r* is the radial coordinate, *R* is the normalized radius of the metasurface, *n* represents the number of polynomial coefficients, and *a_i_* is the optimized phase coefficient.

We used the optical design software ZEMAX to optimize the phase coefficients *a_i_*. In detail, the Binary 2 surface type was used to simulate the phase modulation of metasurfaces. Also, we employed the multiple configuration function in ZEMAX to set opposite diffraction orders of the Binary 2 surface type so that the step-zoom focusing can be achieved. The RMS spot of the focal plane for various configurations and FOVs were used as the evaluation criterion. The optimization process was as follows: First, the system parameters, including entrance diameter, FOV, and working wavelength were set. The effective focal length and working F-number operands were added in the merit function as constraints. Then, we set the back focal length and phase coefficients as optimization variables. Two metasurfaces corresponding to the previously calculated focal lengths were designed. It should be noted that more phase coefficients will improve the system performance in theory, whereas this strategy hardly works as the focusing performance is close to the diffraction limit. Therefore, five phase coefficients were used for a good balance between system performance and optimization efficiency referring to the previous work. Second, the two designed metasurfaces are cemented with the fused silica substrate. In turn, the back focal length, phase coefficients, and substrate thickness were added as optimization variables in order to minimize the RMS focal spot size for different configurations and FOVs. Finally, the optimized substrate thickness is 3.4 μm, and the back focal length is 23.7 μm. The phase coefficients for the phase modulation of the front and back metasurfaces with a normalized radius of 10 μm are presented in Table 1.

**Table 1:** Optimized phase coefficients of the front and back metasurfaces.

	Diameter	*a*_1_	*a*_2_	*a*_3_	*a*_4_	*a*_5_

front metasurface	20 μm	−48.8883	−0.3008	2.1089	0.3695	−0.7718
rear metasurface	30 μm	55.0949	2.7000	−4.2203	1.7344	−0.2596

Now, the double-layer step-zoom metalens was obtained by the optimization process above. This lens can achieve diffraction-limited imaging with two working modes corresponding to different focal lengths.

Referring to a previously reported work [28], if a specially designed super-oscillatory phase is superimposed on the stop pupil surface (i.e., the front metasurface) of this double-layer step-zoom metasurface, the proposed SSL will be realized. The intensity distribution in the image plane of the SSL can be calculated by diffraction propagation methods, such as angular spectrum diffraction. To obtain a specific sub-diffraction focal spot, we developed a multiply constrained optimization model with a single objective. The objective function is defined as the central intensity of the sub-diffraction focal spot with constraints of the full width at half maximum (FWHM) of the focal spot, the maximum relative intensity of the sidelobe within the local field of view, and the super-oscillation phase distribution. The mathematical expression of this model is as follows:


[3] $$\begin{array}{c} {\text{Objective:}}_{{}_{{}_{{}_{}}}}\;\\ \text{Constraints: } \end{array}\begin{array}{c} \max I\left(0\right)\;\\ \text{ FWHM}\leq G\cdot \frac{0.5145\lambda }{\text{NA}}\;\\ \begin{array}{l} \frac{I\left(\rho \right)}{I\left(0\right)}\leq M,\;\frac{0.61\lambda }{\text{NA}}<\rho \leq L\\ \varphi_{\text{so}}\left(r_{i}\right)\in \left\{0,\pi \right\}, \end{array} \end{array}$$

where *G* is the sub-diffraction factor, *M* indicates the ratio between the maximum intensity of the sidelobe in the local region *L* and the central intensity of the sub-diffraction focal spot, ρ is the radial coordinate in the focal plane, and *r_i_* represents the radius of the *i*-th annular ring of the super-oscillatory phase profile. To ensure the optimization efficiency, the super-oscillation phase distribution is divided into *N* rings equally along the radial direction. Each annular ring takes a phase value of 0 or π. In our proposed SSL, the FWHM is set to be 0.84 times of the diffraction limit (calculated by 0.5145λ/NA, *G* is set to be 0.84), and *M* is set to be 0.2. According to the particle swarm optimization algorithm [29], the final radially normalized π-phase-jump positions of the super-oscillation phase are 0.15, 0.2 and 0.4, respectively.

**Figure 2:**
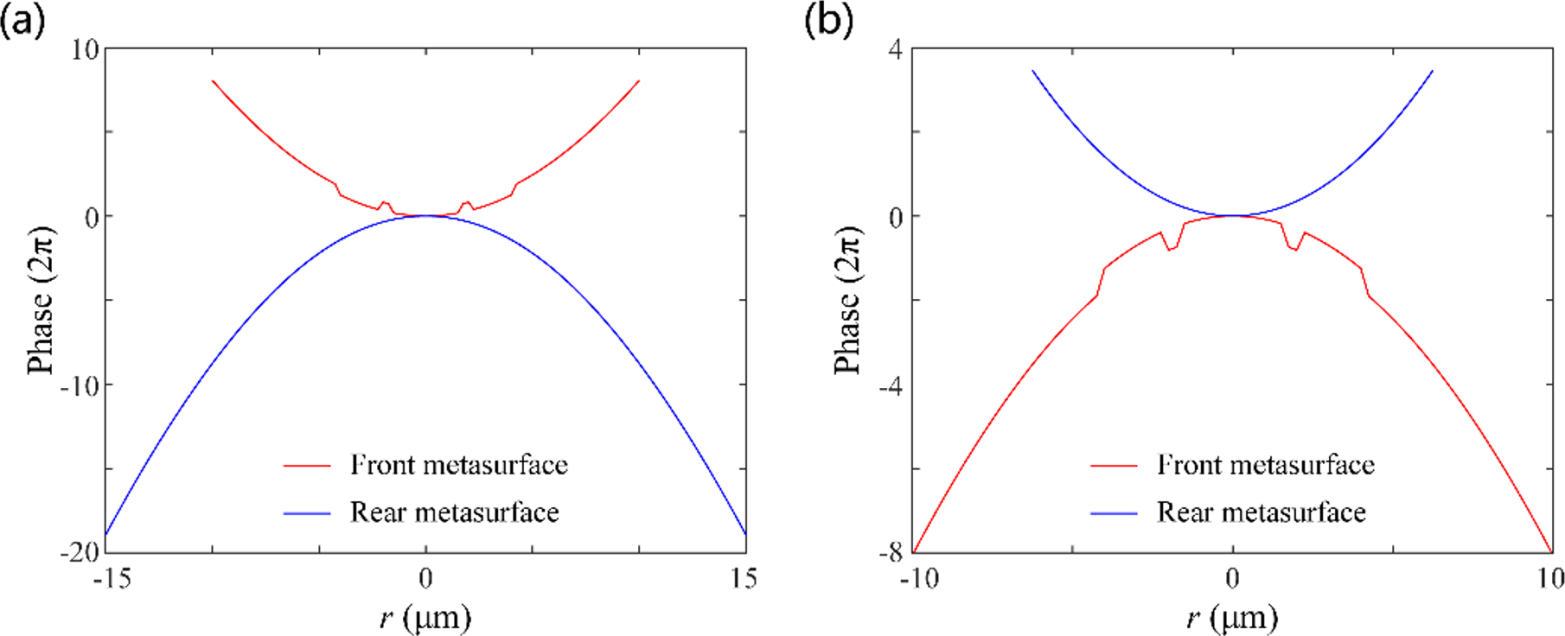
Phase profiles of the front and rear metasurfaces for (a) short and (b) long focal lengths.

The phase modulation of the super-oscillatory step-zoom metalens can be divided in two parts, namely focusing phase and super-oscillatory phase. Phase modulation is achieved by TiO_2_ nanopillars based on the geometric phase, which is inherently polarization-sensitive. The front and rear metasurfaces have the opposite optical power and work as a concave lens and a convex lens for the incident right-handed circularly polarized (RCP) light, or a convex lens and a concave lens for the incident left-handed circularly polarized (LCP) light. For the super-oscillatory phase, it takes the value of 0 or π, so the modulation remains invariant for incident left-handed or right-handed circularly polarized light. It means that the super-oscillatory step-zoom metalens can achieve super-resolution focusing for both left-handed and right-handed circularly polarized light. The proposed SSL can be achieved with the optimization method above. The phase distribution, in turn, of the front metasurfaces is finally obtained by superimposing the optimized super-oscillatory phase. As the LCP light impinges on the SSL, it works with a long focal length. It can switch to the short focal length with incident RCP light. As shown in Figure 2, the front and rear metasurfaces have the opposite optical powers. In this way, we can realize sub-diffraction step-zoom focusing with the designed SSL.

**Figure 3:**
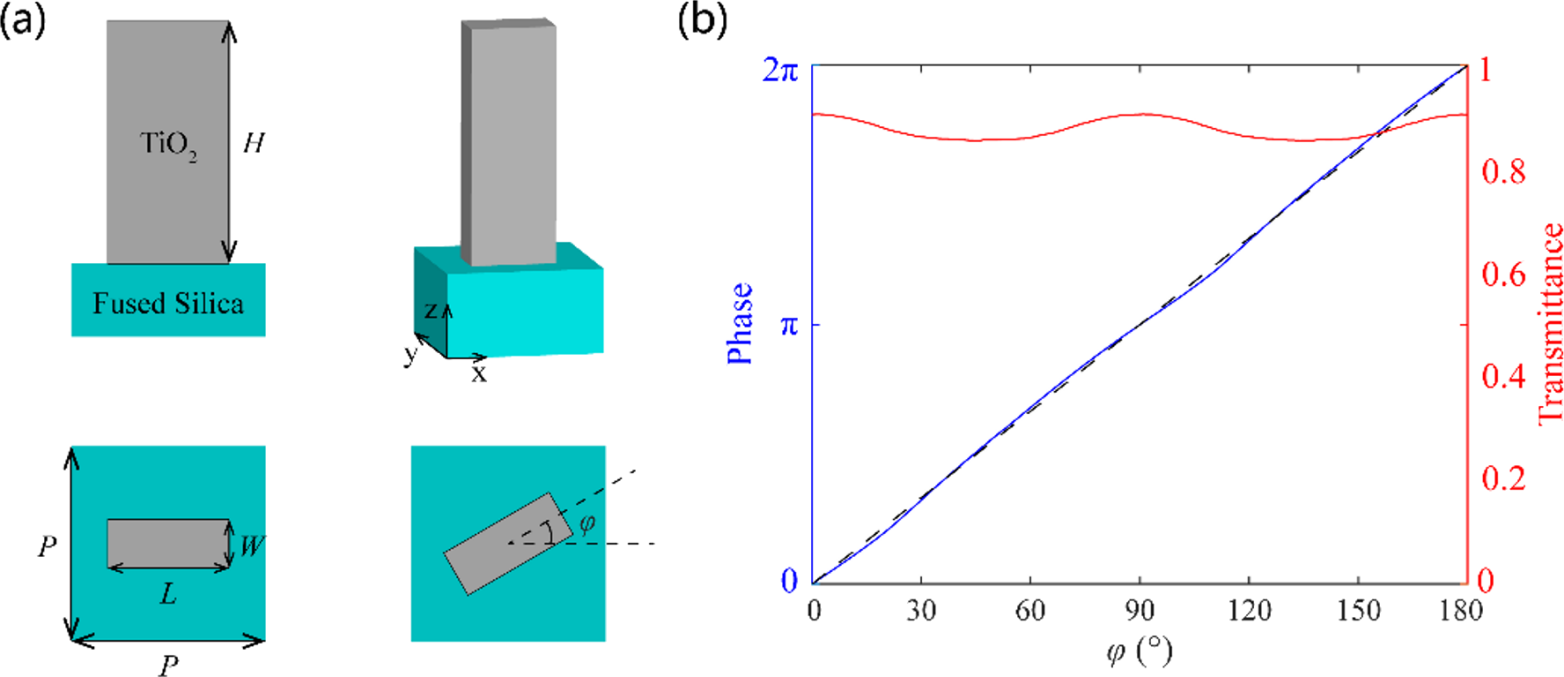
(a) Schematic of a TiO_2_ rectangular nanopillar. (b) Simulated transmittance and phase modulations of the unit cells with different orientations. The black dashed line represents the theoretical modulated phase.

To dynamically modulate the phase distribution, we designed a unit cell structure based on the geometric phase (i.e., Pancharatnam–Berry phase) principle [30–31]. Generally, metasurfaces based on geometric phases are easier to process, and the phase modulation in the whole 2π range can be achieved by simply changing the orientation of the unit cell. Also, the higher the refractive index of the material in the visible band is, the weaker the coupling among the unit cells will be, which leads to a higher efficiency of the metasurface. Thus, rectangular nanopillars composed of titanium dioxide (the refractive index is 2.87 at a wavelength of 632.8 nm) are used as unit cells of the two metasurfaces. The fused silica glass is used as substrate in the SSL. Theoretically, for the metasurface based on the geometric phase principle, the phase modulation of the transmitted RCP light is exactly twice of the orientation of the unit cell under the LCP incidence. The field modulation of the unit cells with various orientations and sizes were numerically simulated by CST Microwave Studio. After a parameter sweep, as illustrated in Figure 3a, the unit cell was selected to obtain high polarization conversion efficiency with length *L* = 200 nm, width *W* = 80 nm, height *H* = 600 nm, and period *P* = 250 nm × 250 nm. It is demonstrated in Figure 3b that the transmittance varies only little for different orientation angles and the modulated phase changes almost linearly with a factor of two, which also proves the geometric phase principle and the excellent performance of the designed unit cells.

## Simulation Results and Performance Analysis

We also use CST to perform the full-wave simulation for the designed SSL, setting the wavelength to 632.8 nm and incident angle of 0°, 1.1°, and 1.6° along the *x*-axis direction. Since the whole simulation area is too large and time-consuming for a calculation, we employed the following method to improve the simulation speed: First, we simulated the interaction between the light and the metasurface structure by CST and extracted the optical field at 0.3 μm from the back surface of the SSL. Then, we utilized the scalar angular spectrum diffraction method to calculate the diffraction propagation behind the extracted field, and thus obtained the optical field distribution at a specific plane.

**Figure 4:**
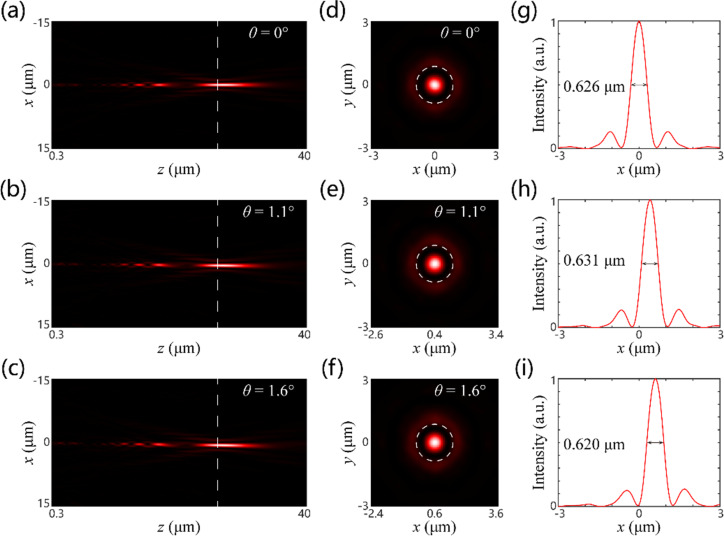
Simulated intensity distributions for different incident angles with RCP incidence corresponding to the SSL with short focal length. (a–c) Intensity distributions along the propagating direction. The dashed white lines represent the focal plane of the SSL. (d–f) Intensity distributions in the focal plane. The dashed white circles represent the diffraction-limited focal spot (Airy disk). (g–i) Intensity distributions of the sub-diffraction spot along the *x*-direction.

**Figure 5:**
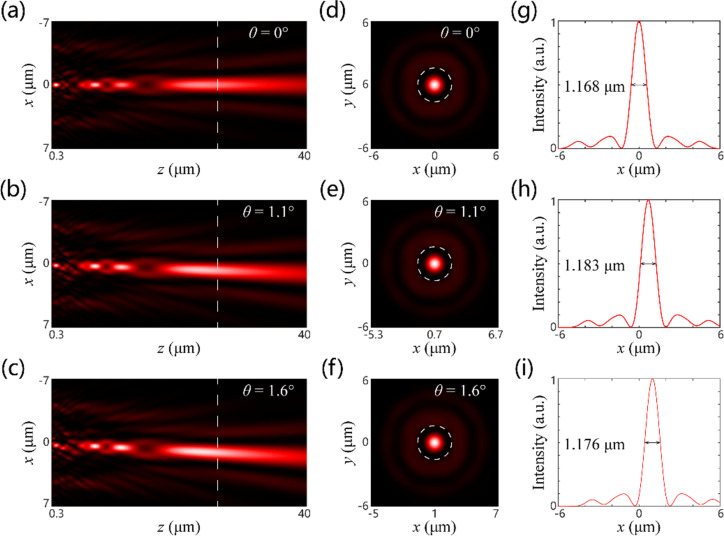
Simulated intensity distributions for different incident angles with LCP incidence corresponding to the SSL with long focal length. (a–c) Intensity distributions along the propagating direction. The dashed white lines represent the focal plane of the SSL. (d–f) Intensity distributions in the focal plane. The dashed white circles represent the diffraction-limited focal spot (Airy disk). (g–i) Intensity distributions of the sub-diffraction spot along the *x*-direction.

As shown in Figure 4 and Figure 5, we calculated the intensity distribution of the optical field along the propagation direction for various polarization states (corresponding to different focal lengths) and incident angles. The sub-diffraction spots are all well formed in the designed image plane for all cases. In Figure 4d–i and Figure 5d–i, the calculated intensity distributions for the image plane of *z* = 23.7 μm are shown. It is clearly indicated that the FWHMs of the sub-diffraction focal spots are very close to the designed value (0.612 and 1.128 μm for the short and long focal lengths, respectively). The difference between the simulation results and the theoretical values is mainly attributed to the insufficient sampling and optical modulation deviation of metasurface. Nevertheless, the simulation results still demonstrate the super-diffraction focusing performance of the SSL for two different focal lengths, which illustrates the effectiveness of our proposed method. The transverse displacements of the sub-diffraction focal spots were calculated for various incident angles, as illustrated in Figure 4g–i and Figure 5g–i. The values are 0, 0.4 and 0.6 μm, and 0, 0.7 and 1 μm, respectively. They are consistent with the theoretical results of 0, 0.38 and 0.56 μm, and 0, 0.77 and 1.12 μm, respectively. In addition, the focusing efficiencies were calculated to be, respectively, 9.98%, 6.45% and 4.99%, and 22.24%, 13.27% and 7.10% for different incident angles. Here, the focusing efficiency is defined as the ratio between the intensity in the main lobe region of the super-diffraction focal spot and the total incident intensity. It is found that the focusing efficiencies for the short focal length of the SSL are all smaller than those for the long focal length of SSL. Also, the focusing efficiency decreases with the increase of the incident angle. This suggests that there is a more pronounced optical aberration of the short focal length than of the long focal length, which corresponds well with the traditional geometric optical theory. It should be noted that the low focusing efficiency of the SSL arises from the redistribution of the optical field energy. This is a typical feature of the optical super-oscillation phenomenon in which the main lobe of the super-diffraction spot is often surrounded by some sidelobes with considerable intensity, leading to the unavoidable energy loss.

## Discussion

For simplification, we did not take into consideration the influence of factors such as the amplitude and phase modulation deviation of the metasurface, which may lead to the degradation of the sub-diffraction focusing performance. The first is the phase modulation error due to discrete sampling. In the design, it is assumed that the physical size of the unit cell could be infinitely small for the convenience of optimization. In fact, a unit cell has a specific size and could only achieve a step-like discrete phase, which, in turn, deviates from the desired phase modulation. Second, the inhomogeneous amplitude modulation also contributes to performance degradation. In our design, it is assumed that only phase modulation with uniform amplitude is achieved by the metasurface. Nonetheless, due to the shadowing effect and coupling phenomenon among unit cells, the transmittance of the unit cells is affected by the incident angle and the orientation of unit cells. Third, the residual co-polarization component could lead to noticeable stray light. As we used a double-layer metasurface based on the geometric phase principle, there are two polarization conversions. For the ideal case, the incident LCP will be converted to RCP, and then converted to LCP again. However, the polarization conversion efficiency cannot reach 100%, which would cause some stray light in the SSL. To address these problems, the influence of discrete sampling errors could be relieved using a catenary structure, while the influence of stray light could be minimized by using a metasurface design based on the propagation phase principle.

In principle, there are always inevitable sidelobes around the sub-diffraction hotspot in the super-oscillatory phenomenon, which are detrimental to imaging and render low efficiency and limited FOV. To solve the problem, some methods have been used in super-oscillatory imaging, such as confocal scanning imaging and specially designed super-oscillatory spots [9–10]. Nevertheless, the low efficiency may be tolerable for some specific applications, such as observing target objects with high illumination intensity in microscopic systems. High-sensitivity detectors and long exposure time would be also advantageous.

## Conclusion

In summary, we have proposed a super-oscillatory step-zoom lens composed of titanium dioxide nanopillars. The device operates at a wavelength of 632.8 nm and has two working modes with long or short focal lengths, corresponding to the telescopic and wide-angle application scenarios, respectively. In the proof-of-principle, the super-oscillatory step-zoom lens can achieve super-resolution focusing in two separate focusing cases. The simulation results show good agreement with the theory. This paper provides a novel method for constructing a dynamically tunable super-oscillatory lens, which has great potential applications in the field of super-resolution microscopic imaging and optical precision machining.
